# Reference Gene Selection for Quantitative PCR Studies in Sheep Neutrophils

**DOI:** 10.3390/ijms140611484

**Published:** 2013-05-30

**Authors:** William R. Vorachek, Hugejiletu, Gerd Bobe, Jean A. Hall

**Affiliations:** 1 Department of Biomedical Sciences, College of Veterinary Medicine, Oregon State University, Corvallis, OR 97331, USA; E-Mail: william.vorachek@oregonstate.edu; 2 Department of Animal and Rangeland Sciences, College of Agricultural Sciences, Oregon State University, Corvallis, OR 97331, USA; E-Mail: gerd.bobe@oregonstate.edu; 3 Linus Pauling Institute, Oregon State University, Corvallis, OR 97331, USA

**Keywords:** blood neutrophils, foot rot, *Ovis aries*, qPCR, reference genes, selenium treatment

## Abstract

Reference genes are essential for studying mRNA expression with quantitative PCR (qPCR). We investigated 11 potential neutrophil reference genes (*RPL19*, *GAPDH*, *ACTB*, *B2M*, *HPRT*, *G6PD*, *TFRC*, *PGK1*, *YWHAZ*, *SDHA* and *GYPC*) for sheep under disease conditions of foot rot (FR) and with or without Se supplementation. Initial screening was based on gene expression level (<28 Cq cycles) and variability (SD < 1.5 Cq cycles) and excluded *TFRC*, *GYPC* and *HPRT* from further analysis. Expression stability of the remaining genes was evaluated using four software programs: geNorm, NormFinder, BestKeeper and the comparative delta Cq method. The neutrophil reference genes, *G6PD*, *YWHAZ*, *GAPDH*, *RPL19* and *SDHA*, consistently ranked among the top five most stable genes under these experimental conditions. The *SDHA* gene expression was not stable in FR-diseased sheep receiving Se treatment and, thus, cannot be recommended as a reference gene. The commonly used genes, *PGK1*, *ACTB* and *B2M*, were not reliable reference genes, underscoring the need to validate neutrophil reference genes under different experimental conditions. Multiple references genes rather than a single gene may provide more robust and reliable results. The best pair of reference genes was *SDHA*/*G6PD* in healthy sheep and *GADPH*/*YWHAZ* in FR-diseased sheep.

## 1. Introduction

Quantitative PCR (qPCR) is a powerful tool for gene expression analysis [1]. Expression data from genes of interest are normalized against reference genes to correct for the initial amount of starting material in order to determine expression differences with disease or in response to treatment. Reference genes, which are often referred to as housekeeping genes, are assumed to be constitutively expressed; however, reference gene expression may vary depending upon the cell type analyzed and experimental conditions [2–5]. Prior to examining expression data from neutrophils in healthy and foot rot (FR) affected sheep, the selection of appropriate reference genes is critical.

Foot rot is a common, contagious bacterial disease of sheep that results in lameness and significant economic losses for sheep producers [6]. We have previously reported that sheep affected with FR have lower whole blood selenium (Se) concentrations and that Se supplementation in conjunction with routine control practices accelerates recovery from FR [7]. Foot rot is caused by infection with the bacterium *Dichelobacter nodosus*, an anaerobic bacterium, in association with other bacteria, particularly *Fusobacterium necrophorum* (reviewed in [7]). The role of the immune system in the etiology of FR is not well understood, and our goal is to understand the mechanism(s) by which Se may facilitate recovery from FR. It is known that Se deficiency inhibits neutrophil functions [8]. In the previously reported study, sheep affected with FR were injected monthly for 15 months with either Se or saline, whereas healthy sheep received no treatment [7]. Before assessing the relative abundance of mRNA for genes associated with neutrophil functions, neutrophil reference genes in healthy and FR-diseased sheep, with and without Se supplementation, are needed.

Other research groups have described neutrophil reference genes in human neutrophils [9,10] and in ovine whole blood [11]. For example, a TATA box binding protein, beta-actin (ACTB), and succinate dehydrogenase complex subunit A (SDHA) were reported to be stably expressed in human neutrophils [9]. In ovine whole blood, researchers reported that hypoxanthine phosphoribosyltransferase I (HPRT) and SDHA were suitable neutrophil reference genes [11].

We investigated 11 potential neutrophil reference genes for sheep (Table 1) under disease conditions of FR and with or without Se supplementation, including: *ACTB*, ribosomal protein L19 (*RPL19*), beta-2-microglobin (*B2M*), glyceraldehyde-3-phosphate dehydrogenase (*GAPDH*), tyrosine 3-monoxygenase/tryptophan 5-monooxygenase activation protein zeta polypeptide (*YWHAZ*), *SDHA*, phosphoglycerate kinase 1 (*PGK1*), glucose-6-phosphate dehydrogenase (*G6PD*), *HPRT*, transferrin receptor (*TFRC*) and glycophorin C (*GYPC*). We assessed gene expression level and analyzed gene stability using the programs geNorm [12], NormFinder [13], BestKeeper [14] and a comparative delta Cq method [15], in neutrophils from healthy sheep, FR-diseased sheep without Se-supplementation, FR-diseased sheep with Se-supplementation and in all groups combined.

## 2. Results and Discussion

### 2.1. Expression Level of Neutrophil Reference Genes Evaluated in This Study

The expression of 11 commonly used reference genes (Table 1) was measured by qPCR experiments. The observed Cq values were distributed over a wide range in all sheep groups (Table 2), including highly expressed *ACTB* (Cq ± SD, 18.63 ± 0.69; Cq range, 2.15) and less transcribed *TFRC* (29.25 ± 1.70; Cq range, 5.54) and *GYPC* (30.04 ± 0.14; Cq range, 2.17). Our results for *ACTB* and *GYPC* are similar to those reported by Peletto [11] for ovine whole blood. The most variation was associated with *HPRT* (24.52 ± 2.22; Cq range, 6.12), which was the only gene that did not pass the test for normal distribution (Shapiro-Wilk W = 0.79; *p* = 0.02), and thus, *HPRT* was excluded from further analysis.

We arbitrarily selected a gene expression level >28 cycles or high variability (SD > 1.5 cycle) for exclusion of potential reference genes from further consideration. This eliminated *TFRC*, *GYPC* and *HPRT* from further analysis. Our rationale was that the delta Cq for genes of interest compared to reference genes in subsequent studies would be more accurate if reference genes were expressed in sufficient copy numbers to be reliably detected in all samples and have limited variation.

### 2.2. GeNorm Analysis of Reference Genes

The program geNorm [12] provides a measure of gene expression stability by calculating the average pairwise variation of each reference gene from all the other reference gene candidates. In addition, it performs a ranking of the candidate genes by stepwise exclusion of the worst scoring gene and repeated recalculation of the average gene expression stability value. The designers of geNorm also stipulate that neither experimental conditions nor cell type affects the expression ratio of a true reference gene pair. This is based on the premise that the expression ratio of reference genes should be the same in all experimental samples. Hence, expression ratios of gene pairs were used as a measure of reference gene stability. The stability values calculated by geNorm were used to rank gene expression in our study for potential neutrophil reference genes (Table 3).

The lower the stability value, the more likely a candidate gene will be useful as a reference gene. Low stability values indicate stable gene expression [12]. We arbitrarily selected a gene stability level <0.5 for inclusion of potential reference genes for further consideration. Based on geNorm analysis, *GAPDH* (Cq range, 1.34), *YWHAZ* (Cq range, 1.13) and *G6PD* (Cq range, 0.79) would be suitable as reference genes in healthy, FR-diseased sheep or FR-diseased sheep treated with Se. In addition, *SDHA* (Cq range, 1.20) and *RPL19* (Cq range, 1.96) had gene stability values that were <0.5, justifying their use, as well. Three of the candidate neutrophil reference genes would be excluded from consideration: *PGK1* (Cq range, 1.69), *ACTB* (Cq range, 2.15) and *B2M* (Cq range, 4.66). In addition, *ACTB* (*p* = 0.04) and *B2M* (*p* = 0.03) were the only two genes that differed significantly (*p* ≤ 0.05) among the three groups; specifically, FR-sheep with and without Se administration differed in *ACTB* (*p* = 0.01) and *B2M* Cq values (*p* = 0.009). In ovine whole blood studies, *B2M* was also outperformed by other genes as a suitable reference gene [11].

Using multiple reference genes rather than a single reference gene is likely to provide more robust and reliable results [12]. In the geNorm algorithm, the optimal number of reference genes is determined when the addition of a further gene leads to a negligible reduction in the average of gene stability estimates. In our study, this was reached with two reference genes in each of the sheep categories. For healthy control sheep, the best pair of reference genes was *SDHA*/*G6PD*, whereas for FR-diseased sheep, regardless of whether they received Se treatment, the best pair of reference genes was *GADPH*/*YWHAZ*.

### 2.3. NormFinder Analysis of Reference Genes

The NormFinder program uses a model-based approach that also estimates the variation between sample subgroups. The program analyzes inter- and intra-group expression variation of potential reference genes. A stability value is calculated based on analysis of gene expression data, and the potential reference genes are then ranked. Lower values are assigned to the most stable genes. When NormFinder was used to identify potential neutrophil reference genes in this study, *G6PD*, *GAPDH*, *YWHAZ* and *RPL19* ranked as the best choices (Table 4). The *SDHA* gene did not rank as high in FR-diseased sheep treated with Se. Similar to geNorm analysis, three of the candidate neutrophil reference genes would be excluded from consideration, as they ranked consistently at or near the bottom of the rank order: *PGK1*, *ACTB* and *B2M*.

### 2.4. BestKeeper Analysis of Reference Genes

The program BestKeeper estimates the expression stability by performing a pairwise correlation analysis of each pair of candidate gene Cq values. It then calculates the geometric mean of the best suited genes. The weighted index is correlated with up to ten target genes using the same pairwise correlation analysis. When BestKeeper was used to find potential reference genes (Table 5), *G6PD*, *YWHAZ*, *SDHA* and *GAPDH* performed well in healthy sheep and in FR-diseased sheep that were not treated with Se. After Se treatment of FR-diseased sheep, *SDHA* and *GAPDH* were near the bottom of the rank order. Similar to geNorm and NormFinder analyses, three of the candidate neutrophil reference genes would be excluded from consideration, as they ranked consistently at or near the bottom of the rank order: *PGK1*, *ACTB* and *B2M*.

### 2.5. Delta Cq Analysis of Reference Genes

Finally, a delta Cq analysis [15] was performed; this analysis is similar to the geNorm program in that pairs of genes are compared using Cq differences. The comparative delta Cq method compares the Cq value differences between two reference genes from different samples, and if the delta Cq value between pairs of genes remains constant for all samples tested, then those reference genes are either stably expressed or co-regulated. Potential reference gene candidates were compared in this study and ranked based on Cq value differences to determine those with the least variance. Results are shown in Table 6. The best choices for neutrophil reference genes were *G6PD*, *YWHAZ*, *GAPDH* and *RPL19*. Again, *SDHA* performed well in healthy sheep and FR-diseased sheep that were not treated with Se, but after Se treatment of FR-diseased sheep, it ranked near the bottom of the rank order. As for geNorm, NormFinder and BestKeeper analyses, three of the candidate neutrophil reference genes would be excluded as reference genes from consideration, as they ranked consistently at or near the bottom of the rank order: *PGK1*, *ACTB* and *B2M*.

### 2.6. Stability of Neutrophil Reference Genes Evaluated in This Study

The neutrophil reference genes, *G6PD*, *YWHAZ*, *GAPDH*, *RPL19* and *SDHA*, were consistently ranked among the top five most stable genes by all four programs (Table 7). The reference gene *SDHA* was ranked lower after Se treatment of FR-diseased sheep in three of the program analyses and would, therefore, not be the best choice as a reference gene. Several conventional reference genes proved to be less reliable, including *PGK1*, *ACTB* and *B2M*. These results underscore the need to validate neutrophil reference genes under different experimental conditions.

Vandesompele *et al.* [12] suggested that normalization based on multiple references genes rather than a single gene is likely to provide more robust and reliable results. Using the geNorm algorithm, in healthy control sheep, the best pair of reference genes was *SDHA*/*G6PD*, whereas for FR-diseased sheep, regardless of whether they received Se treatment, the best pair of reference genes was *GADPH*/*YWHAZ*.

In ovine whole blood, *SDHA* and *YWHAZ* were found to be good reference genes that were not affected by disease status and heat stress conditions; the geometric mean of these two stable genes was an accurate normalization factor [11]. In the same study, *GAPDH* was the gene with the highest degree of individual variation in expression stability according to the geNorm algorithm, yet it ranked as the second most stable gene in the disease-stressed sheep group [11]. Similar to our results, Taylor *et al.* [16] found that *GAPDH* was one of the most stable genes tested in ovine peripheral blood mononuclear cells during infection with *Mycobacterium avium* subsp. *paratuberculosis*, although the study was based on analysis of the standard deviation of Cq values and not on geNorm analysis.

## 3. Experimental Section

### 3.1. Candidate Genes for Expression Studies

Eleven genes were selected from commonly used reference genes [10], *transferrin receptor* (*TFRC*), *phosphoglycerate kinase 1* (*PGK1*), *glycophorin C* (*GYPC*), *YWHAZ*, *SDHA*, *GAPDH*, *G6PD*, *ACTB, B2M*, *RPL19* and *HPRT*. The length of the primers ranged from 18 bp to 23 bp, GC content varied from 45% to 60% and the expected PCR product size was 71 bp to 126 bp (Table 8). All primers were synthesized by Sigma Aldrich (St. Louis, MO, USA) and purified by desalting.

### 3.2. Whole-Blood Collection

All sheep were bled at the end of the 15-month treatment period [7]. The experimental protocol was reviewed and approved by the Oregon State University Animal Care and Use Committee. Jugular venous blood was collected into evacuated ethylenediaminetetraacetic acid (EDTA) tubes (10 mL; final EDTA concentration 2 g/L; Becton Dickinson, Franklin Lakes, NJ, USA) and stored on ice until further processing for neutrophil isolation. Blood was transported on ice to the lab after collection.

### 3.3. Neutrophil Isolation

Neutrophils were isolated from anticoagulated blood within 4 h of collection, using a Percoll gradient technique [17], and then re-suspended in 1× Hank’s balanced saline solution (HBSS, Life Technologies, Grand Island, NY, USA) plus 0.5% FBS (Life Technologies, *Id.*). Cells were counted using a Coulter Counter (Beckman Coulter, Indianapolis, IN, USA). Briefly, 10 mL of anticoagulated blood was centrifuged in a TJ-6 swinging bucket centrifuge (Beckman, *Id.*) at 1000× *g* for 20 min at 22 °C in 50 mL centrifuge tubes (Thermo Fisher Scientific, Waltham, MA, USA). The plasma, buffy coat and one-third of the red blood cell (RBC) pack from each tube were aseptically removed. The remaining RBC packs were mixed with 34 mL ice-cold PBS (Life Technologies, *Id.*) and layered onto 10 mL of freshly prepared 1.084 g/mL Percoll (Sigma-Aldrich, St. Louis, MO, USA). Tubes were centrifuged at 400× *g* for 40 min at 22 °C; all layers above the RBC layer, including the supernatant, mononuclear cell layer and Percoll, were aspirated and discarded. The RBC and neutrophils pelleted at the bottom of the tubes. The RBC were lysed using 24 mL ice-cold hypotonic lysis buffer (10.56 mM Na_2_HPO_4_, 2.67 mM NaH_2_PO_4_, pH 7.3) for 90 s, and then, 12 mL ice-cold hypertonic restore buffer (10.56 mM Na_2_HPO_4_, 2.67 mM NaH_2_PO_4_, 0.43 M NaCl pH 7.3) was added to stop lysis. The RBC lysed solution was centrifuged at 800× *g* for 5 min at 22 °C in a TJ-6 centrifuge. The lysis solution was decanted, and the neutrophils were resuspended and washed twice more with 1× HBSS plus 0.5% FBS. Neutrophils were finally resuspended in 0.25 mL of 1× HBSS plus 0.5% FBS and stored on ice until needed. A 20 μL aliquot of cell suspension was used to determine cell concentration using a Coulter Counter (Beckman, *Id.*). Another 5 μL aliquot was used to assess the purity of neutrophil preparations (differential cell count) by microscopic examination after Wright-Giemsa staining (95% ± 1%; mean ± SEM). The remaining neutrophils were pelleted by centrifugation at 700× *g* for 10 min at 4 °C in a TJ-6 swinging bucket rotor (Beckman, *Id.*); the supernatant was removed, and cells were frozen at −80 °C.

### 3.4. RNA Isolation

Total RNA was extracted within 48 h of neutrophil isolation and freezing at −80 °C, using an RNeasy spin column (RNeasy^®^ Mini Kit; Qiagen Sciences; Gemantown, MD, USA) and following the manufacturer’s instructions. Genomic DNA was eliminated with an RNase-Free DNase (Qiagen Sciences, *Id.*). Total isolated RNA was quantified using an ND-1000 NanoDrop Spectrophotometer (Thermo Fisher Scientific; Waltham, MA, USA). Only samples with A_260_/A_280_ ratios between 1.80 and 2.00 were analyzed further. Samples were stored at −80 °C until qPCR was performed.

### 3.5. Quantitative PCR

The expression level of references genes in neutrophils was determined by qPCR. First strand cDNA was synthesized from 200 ng of total RNA from each sample using the High Capacity cDNA Reverse Transcription Kit (Applied Biosystems, Foster City, CA, USA) and stored at −20 °C. Subsequent qPCR was conducted using RT^2^ SYBR Green qPCR Mastermix (Qiagen Sciences, *Id.*) in 96-well microtiter plates (Applied Biosystems, *Id.*) with a final reaction volume of 25 μL. Each well contained 12.5 μL of qPCR Mastermix, 1 μL of each primer, 1 μL of cDNA sample and 9.5 μL of PCR-grade water. The abundance of gene transcripts was determined by absolute qPCR using the 7300 Real-time PCR System (Applied Biosystems, *Id.*). A negative control (containing all reagents, except target DNA) was included to verify the absence of contamination in each qPCR assay. Each reaction consisted of the following steps: 10-min initial denaturation at 95 °C to activate the polymerase, followed by 40 cycles of 15 s denaturation at 95 °C and 1 min annealing-elongation at 60 °C.

### 3.6. Statistical Analysis of Neutrophil Gene Expression Stability

The Cq values were reported as the mean, standard deviation (SD) and range. The expression stability of potential neutrophil reference genes was evaluated using generally accepted Excel-based software tools [18], according to instructions provided by the program developers, *i.e.*, geNorm [12], NormFinder [13], BestKeeper [14] and a comparative delta Cq method [15]. Ranking of potential neutrophil reference genes was performed for neutrophils collected from healthy sheep, FR-diseased sheep untreated with Se, FR-diseased sheep treated with Se and all groups combined. To provide a summary statistic for the four algorithms, the geometric mean of the ranking for each of the four algorithms was calculated [19]; the gene with the lowest value was viewed as the most stable reference gene. Normality of gene expression was tested using the Shapiro Wilk statistic in SAS, version 9.2 (SAS, Inc., Cary, NC, USA), software. Group averages of Cq values were compared overall, between healthy and FR-diseased sheep and between FR-diseased sheep with and without Se-administration using analysis of variance methods (PROC GLM). Statistical significance was declared at *p* ≤ 0.05.

## 4. Conclusions

A literature search for candidate reference genes in the species and cell type of interest provides a starting point for gene selection. Once potential reference genes have been screened for expression level and overall variability, candidate genes can be further analyzed for expression stability using four readily available software algorithms: geNorm, NormFinder, BestKeeper and the comparative delta Cq method. The neutrophil reference genes, *G6PD*, *YWHAZ*, *GAPDH*, *RPL19* and *SDHA*, consistently ranked among the top five most stable genes under our experimental conditions. The reference gene *SDHA* ranked lower after Se treatment of FR-diseased sheep in three of the program analyses and would, therefore, not be our best choice as a reference gene. Several conventional reference genes proved to be less reliable, including *PGK1*, *ACTB* and *B2M*. These results underscore the need to validate neutrophil reference genes under different experimental conditions. Multiple references genes rather than a single gene may provide more robust and reliable results. In healthy control sheep, the best pair of reference genes was *SDHA*/*G6PD*, whereas for FR-diseased sheep, regardless of whether they received Se treatment, the best pair of reference genes was *GADPH*/*YWHAZ*.

## Supplementary Information



## Figures and Tables

**Table 1:** Candidate reference genes evaluated in this study.

	Gene name	Function	Accession number *	Gene synonyms
*ACTB*	*Beta-actin*	Cytoskeletal structural protein	NM_001009784.1	Actin cytoplasmic 1; beta-actin
*RPL19*	*Ribosomal protein L19*	Found in the large ribosomal subunit	XM_004012836.1	
*B2M*	*Beta-2-microglobin*	Beta-chain of class I major histocompatibility complex (MHC) molecules	NM_001009284.2	
*GAPDH*	*Glyceraldehyde-3-phosphate dehydrogenase*	Enzyme in carbohydrate metabolism	NM_001190390.1	GAPD; G3PDH
*YWHAZ*	*Tyrosine* *3-monoxygenase/tryptophan* *5-monooxygenase activation* *protein, zeta polypeptide*	Signal transduction	NM_001267887.1	14-3-3 protein zeta/delta; tyrosine 3-monooxygenase
*SDHA*	*Succinate dehydrogenase complex, subunit A*	Mitochondrial respiratory chain	XM_004017097.1	
*PGK1*	*Phosphoglycerate kinase I*	Catalyzes the transfer of the high-energy phosphate group of 1,3-bisphosphoglycerate to ADP, forming ATP and 3-phosphoglycerate	NM_001142516.1	
*G6PD*	*Glucose-6-phosphate dehydrogenase*	Enzyme in carbohydrate metabolism	NM_001093780.1	
*HPRT*	*Hypoxanthine phosphoribosyl-transferase 1*	Phosphoribosyl-transferase (PRT)-type I domain	XM_004022693.1	HPRT1
*TFRC*	*Transferrin receptor*	Protease-associated domain containing proteins, like transferrin receptor	XM_004003001.1	p90; CD71
*GYPC*	*Glycophorin C*	Integral RBC membrane binding protein	XM_004004772.1	BOS_1916; CD236; CD236R

* Ref Seq: NCBI Reference Sequence database http://www.ncbi.nlm.nih.gov/RefSeq.

**Table 2:** Individual Cq values of the candidate reference genes in healthy control and foot rot-affected sheep, with or without selenium (Se) treatment, and combined groups.

Gene symbol	Healthy control sheep (*n* = 4)		Foot rot-diseased sheep, untreated with Se (*n* = 6)		Foot rot-diseased sheep, treated with Se (*n* = 6)		Combined groups (*n* = 16)	
			
	Mean Cq	SD	Mean Cq	SD	Mean Cq	SD	Mean Cq	SD
*ACTB*	18.68	0.88	18.14	0.52	19.09	0.36	18.63	0.69
*RPL19*	18.72	0.56	18.68	0.41	19.22	0.35	18.89	0.49
*B2M*	19.61	1.47	19.01	0.80	20.72	0.87	19.80	1.18
*GAPDH*	20.09	0.39	19.59	0.28	19.88	0.45	19.82	0.38
*YWHAZ*	20.82	0.35	20.48	0.33	20.60	0.37	20.61	0.35
*SDHA*	20.99	0.39	21.06	0.30	21.25	0.45	21.11	0.34
*PGK1*	21.12	0.51	21.24	0.53	21.58	0.41	21.34	0.51
*G6PD*	21.82	0.23	21.62	0.22	21.95	0.24	21.80	0.26
*HPRT*	25.54	2.33	23.76	1.86	24.59	2.07	24.52	2.22
*TFRC*	29.17	1.82	28.02	1.04	30.55	0.89	29.25	1.70
*GYPC*	29.56	0.59	30.24	0.19	30.15	0.71	30.04	0.55

**Table 3:** Stability ranking of candidate reference genes in healthy control and foot rot-affected sheep, with or without selenium (Se) treatment, and in combined groups, by the geNorm algorithm (lower stability values indicate more stable gene expression).

Healthy control sheep (*n* = 4)		Foot rot-diseased sheep, untreated with Se (*n* = 6)		Foot rot-diseased sheep, treated with Se (*n* = 6)		Combined groups (*n* = 16)	

Gene Symbol and Stability Value							
*SDHA|G6PD*	0.041	*GAPDH|YWHAZ*	0.235	*GAPDH|YWHAZ*	0.195	*GAPDH|YWHAZ*	0.278
*YWHAZ*	0.258	*G6PD*	0.253	*G6PD*	0.265	*G6PD*	0.307
*GAPDH*	0.333	*SDHA*	0.284	*SDHA*	0.288	*SDHA*	0.327
*RPL19*	0.449	*RPL19*	0.346	*RPL19*	0.326	*RPL19*	0.396
*PGK1*	0.542	*PGK1*	0.426	*ACTB*	0.420	*PGK1*	0.478
*ACTB*	0.631	*ACTB*	0.495	*PGK1*	0.466	*ACTB*	0.546
*B2M*	0.756	*B2M*	0.588	*B2M*	0.583	*B2M*	0.683

**Table 4:** Stability ranking of candidate reference genes in healthy control and foot rot-affected sheep, with or without selenium (Se) treatment and in combined groups, by the NormFinder algorithm (lower stability values indicate more stable gene expression).

Healthy control sheep (*n* = 4)		Foot rot-diseased sheep, untreated with Se (*n* = 6)		Foot rot-diseased sheep, treated with Se (*n* = 6)		Combined groups (*n* = 16)	

Gene Symbol and Stability Value							
*G6PD*	0.253	*G6PD*	0.127	*RPL19*	0.183	*G6PD*	0.198
*SDHA*	0.265	*GAPDH*	0.209	*YWHAZ*	0.265	*RPL19*	0.234
*RPL19*	0.297	*YWHAZ*	0.249	*G6PD*	0.298	*GAPDH*	0.377
*GAPDH*	0.434	*RPL19*	0.281	*ACTB*	0.333	*YWHAZ*	0.406
*YWHAZ*	0.528	*SDHA*	0.379	*GAPDH*	0.360	*SDHA*	0.433
*ACTB*	0.607	*ACTB*	0.474	*PGK1*	0.383	*ACTB*	0.465
*PGK1*	0.779	*PGK1*	0.698	*SDHA*	0.536	*PGK1*	0.594
*B2M*	1.078	*B2M*	0.828	*B2M*	0.897	*B2M*	1.043

**Table 5:** Stability ranking of candidate reference genes in healthy control and foot rot-affected sheep, with or without selenium (Se) treatment, and in combined groups, by the BestKeeper algorithm (lower stability values indicate more stable gene expression).

Healthy control sheep (*n* = 4)		Foot rot-diseased sheep, untreated with Se (*n* = 6)		Foot rot-diseased sheep, treated with Se (*n* = 6)		Combined groups (*n* = 16)	

Gene Symbol and Stability Value							
*GAPDH*	0.131	*G6PD*	0.153	*G6PD*	0.202	*G6PD*	0.226
*SDHA*	0.158	*GAPDH*	0.211	*RPL19*	0.249	*YWHAZ*	0.282
*G6PD*	0.168	*YWHAZ*	0.220	*ACTB*	0.251	*SDHA*	0.291
*YWHAZ*	0.218	*SDHA*	0.220	*YWHAZ*	0.263	*GAPDH*	0.308
*PGK1*	0.388	*RPL19*	0.336	*PGK1*	0.321	*RPL19*	0.380
*RPL19*	0.465	*PGK1*	0.355	*SDHA*	0.346	*PGK1*	0.392
*ACTB*	0.642	*ACTB*	0.377	*GAPDH*	0.354	*ACTB*	0.577
*B2M*	0.951	*B2M*	0.546	*B2M*	0.557	*B2M*	0.906

**Table 6:** Stability ranking of candidate reference genes in healthy control and foot rot-affected sheep, with or without selenium (Se) treatment, and in combined groups, by the comparative delta Cq method (lower stability values indicate more stable gene expression).

Healthy control sheep (*n* = 4)		Foot rot-diseased sheep, untreated with Se (*n* = 6)		Foot rot-diseased sheep, treated with Se (*n* = 6)		Combined groups (*n* = 16)	

Gene Symbol and Stability Value							
*G6PD*	0.58	*G6PD*	0.44	*YWHAZ*	0.47	*G6PD*	0.53
*SDHA*	0.58	*GAPDH*	0.48	*RPL19*	0.49	*RPL19*	0.58
*RPL19*	0.66	*YWHAZ*	0.49	*G6PD*	0.50	*GAPDH*	0.59
*GAPDH*	0.68	*RPL19*	0.53	*GAPDH*	0.52	*YWHAZ*	0.60
*YWHAZ*	0.72	*SDHA*	0.54	*ACTB*	0.56	*SDHA*	0.61
*ACTB*	0.80	*ACTB*	0.62	*PGK1*	0.58	*ACTB*	0.70
*PGK1*	0.90	*PGK1*	0.75	*SDHA*	0.61	*PGK1*	0.76
*B2M*	1.13	*B2M*	0.87	*B2M*	0.93	*B2M*	1.09

**Table 7:** Stability ranking of candidate reference genes in healthy control and foot rot-affected sheep, with or without selenium (Se) treatment, and in combined groups, by the geomean of ranking values for geNorm, NormFinder, BestKeeper and the comparative delta Cq method (lower geomean values indicate more stable gene expression).

Healthy control sheep (*n* = 4)		Foot rot-diseased sheep, untreated with Se (*n* = 6)		Foot rot-diseased sheep, treated with Se (*n* = 6)		Combined groups (*n* = 16)	

Gene Symbol and Geomean Ranking Value							
*G6PD*	1.46	*G6PD*	1.32	*YWHAZ*	1.86	*G6PD*	1.32
*SDHA*	1.86	*GAPDH*	1.86	*RPL19*	2.11	*YWHAZ*	2.63
*GAPDH*	2.83	*YWHAZ*	2.52	*G6PD*	2.28	*GAPDH*	2.71
*RPL19*	4.05	*RPL19*	4.47	*GAPDH*	3.81	*RPL19*	3.16
*YWHAZ*	4.16	*SDHA*	4.47	*ACTB*	4.53	*SDHA*	4.16
*PGK1*	6.19	*PGK1*	6.48	*PGK1*	5.73	*PGK1*	6.48
*ACTB*	6.48	*ACTB*	6.48	*SDHA*	5.86	*ACTB*	6.48
*B2M*	8.00	*B2M*	8.00	*B2M*	8.00	*B2M*	8.00

**Table 8:** Details about primers of the candidate reference genes used for qPCR analysis *.

Gene name	Primer sequences (forward/reverse)	Spanned exons	Amplicon size (bp)
*ACTB*	CCAACCGTGAGAAGATGACC	2nd	97
	CCAGAGGCGTACAGGGACAG	3rd	
*GAPDH*	CTGGCCAAGGTCATCCAT	7th	86
	ACAGTCTTCTGGGTGGCAGT	8th	
*SDHA*	CATCCACTACATGACGGAGCA	4th	90
	ATCTTGCCATCTTCAGTTCTGCT	5th	
*GYPC*	ATCAACATCGCTGTCATTGC	3rd	117
	CTCGTTGGTGTCCTATGTGC	4th	
*RPL19*	AGCCTGTGACTGTCCATTCC	2nd	126
	ACGTTACCTTCTCGGGCATT	3rd	
*YWHAZ*	AGACGGAAGGTGCTGAGAAA	2nd	123
	CGTTGGGGATCAAGAACTTT	3rd	
*PGK1*	ACTCCTTGCAGCCAGTTGCT	3rd	101
	AGCACAAGCCTTCTCCACTTCT	4th	
*HPRT*	CCACCCATCTCCTTCATCAC	4th	71
	TTCTGGGCAGACCTCAAATC	5th	
*TFRC*	TTCTGGGCAGACCTCAAATC	4th	106
	CAGCTTCACGTGGGACATAA	5th	
*G6PD*	TGACCTATGGCAACCGATACAA	10th	76
	CCGCAAAAGACATCCAGGAT	11th	
*B2M*	CTGTCGCTGTCTGGACTGG	1st	86
	TTTGGCTTTCCATCTTCTGG	2nd	

* See additional information about primers, including PCR efficiencies [11].
