# Supplementary material for: Reference Gene Selection for Quantitative PCR Studies in Sheep Neutrophils
Source: Int J Mol Sci. 2013 May 30;14(6):11484–95. doi: 10.3390/ijms140611484 (PMC3709743; doi:10.3390/ijms140611484)
Supplement: Supplementary file 1 [file ijms-14-11484-s001.pdf]

Supplementary Information

Table S1. MIQE checklist for authors, reviewers, and editors (see reference [18]). [1]

	IMPORTANCE	Yes/No/NA(not applicable)
**Experimental design**		
Definition of experimental and control groups	E	Yes
Number within each group	E	Yes
Assay carried out by the core or investigator's laboratory?	D	Yes
Acknowledgment of authors' contributions	D	Yes
**Sample**		
Description	E	Yes
Volume/mass of sample processed	D	Yes
Microdissection or macrodissection	E	NA
Processing procedure	E	Yes
If frozen, how and how quickly?	E	Yes
If fixed, with what and how quickly?	E	NA
Sample storage conditions and duration (especially for FFPE [2] samples)	E	Yes
**Nucleic acid extraction**		
Procedure and/or instrumentation	E	Yes
Name of kit and details of any modifications	E	Yes
Source of additional reagents used	D	Yes
Details of DNase or RNase treatment	E	Yes
Contamination assessment (DNA or RNA)	E	Yes
Nucleic acid quantification	E	Yes
Instrument and method	E	Yes
Purity (A_{260}/A_{280})	D	Yes
Yield	D	No
RNA integrity: method/instrument	E	No
RIN/RQI or C_q of 3' and 5' transcripts	E	NA
Electrophoresis traces	D	NA
Inhibition testing (C_q dilutions, spike, or other)	E	Yes
**Reverse transcription**		
Complete reaction conditions	E	Yes
Amount of RNA and reaction volume	E	Yes
Priming oligonucleotide (if using GSP) and concentration	E	NA
Reverse transcriptase and concentration	E	Yes
Temperature and time	E	Yes
Manufacturer of reagents and catalogue numbers	D	Yes
C_qs with and without reverse transcription	D [3]	No
Storage conditions of cDNA	D	Yes

Table S1. *Cont.*

	IMPORTANCE	Yes/No/NA(not applicable)
**qPCR target information**		
Gene symbol	E	Yes
Sequence accession number	E	Yes
Location of amplicon	D	Yes
Amplicon length	E	Yes
In silico specificity screen (BLAST, and so on)	E	Yes
Pseudogenes, retropseudogenes, or other homologs?	D	NA
Sequence alignment	D	No
Secondary structure analysis of amplicon	D	No
Location of each primer by exon or intron (if applicable)	E	Yes
What splice variants are targeted?	E	NA
**qPCR oligonucleotides**		
Primer sequences	E	Yes
RTPrimerDB identification number	D	No
Probe sequences	D [4]	NA
Location and identity of any modifications	E	NA
Manufacturer of oligonucleotides	D	Yes
Purification method	D	Yes
**qPCR protocol**		
Complete reaction conditions	E	Yes
Reaction volume and amount of cDNA/DNA	E	Yes
Primer, (probe), Mg^{2+}, and dNTP concentrations	E	Yes
Polymerase identity and concentration	E	Yes
Buffer/kit identity and manufacturer	E	Yes
Exact chemical composition of the buffer	D	Yes
Additives (SYBR Green I, DMSO, and so forth)	E	Yes
Manufacturer of plates/tubes and catalog number	D	Yes
Complete thermocycling parameters	E	Yes
Reaction setup (manual/robotic)	D	Yes
Manufacturer of qPCR instrument	E	Yes
**qPCR validation**		
Evidence of optimization (from gradients)	D	Yes
Specificity (gel, sequence, melt, or digest)	E	NA (see ref [11])
For SYBR Green I, C_q of the NTC	E	Yes (not published)
Calibration curves with slope and *y* intercept	E	Yes (not published)
PCR efficiency calculated from slope	E	Yes (not published)
CIs for PCR efficiency or SE	D	No
r^2 of calibration curve	E	Yes (not published)
Linear dynamic range	E	NA
C_q variation at LOD	E	NA
CIs throughout range	D	NA
Evidence for LOD	E	NA
If multiplex, efficiency and LOD of each assay	E	NA

Table S1. *Cont.*

	IMPORTANCE	Yes/No/NA(not applicable)
**Data analysis**		
qPCR analysis program (source, version)	E	Yes
Method of C_q determination	E	Yes
Outlier identification and disposition	E	Yes
Results for NTCs	E	Yes
Justification of number and choice of reference genes	E	Yes
Description of normalization method	E	Yes
Number and concordance of biological replicates	D	Yes
Number and stage (reverse transcription or qPCR) of technical replicates	E	NA
Repeatability (intraassay variation)	E	Yes
Reproducibility (interassay variation, CV)	D	Yes
Power analysis	D	Yes
Statistical methods for results significance	E	Yes
Software (source, version)	E	Yes
C_q or raw data submission with RDML	D	Yes (not published)

[1] All essential information (E) must be submitted with the manuscript. Desirable information (D) should be submitted if available. If primers are from RTPrimerDB, information on qPCR target, oligonucleotides, protocols, and validation is available from that source; [2] FFPE, formalin-fixed, paraffin-embedded; RIN, RNA integrity number; RQI, RNA quality indicator; GSP, gene-specific priming; dNTP, deoxynucleoside triphosphate; [3] Assessing the absence of DNA with a no–reverse transcription assay is essential when first extracting RNA. Once the sample has been validated as DNA free, inclusion of a no–reverse transcription control is desirable but no longer essential; [4] Disclosure of the probe sequence is highly desirable and strongly encouraged; however, because not all vendors of commercial predesigned assays provide this information, it cannot be an essential requirement. Use of such assays is discouraged.

© 2013 by the authors; licensee MDPI, Basel, Switzerland. This article is an open access article distributed under the terms and conditions of the Creative Commons Attribution license (http://creativecommons.org/licenses/by/3.0/).
